# Insight into the epidemiology of infective endocarditis in Portugal: a contemporary nationwide study from 2010 to 2018

**DOI:** 10.1186/s12872-021-01937-3

**Published:** 2021-03-16

**Authors:** Catarina Sousa, Paulo Nogueira, Fausto J. Pinto

**Affiliations:** 1 Centro Cardiovascular da Universidade de Lisboa (CCUL), Centro Académico de Medicina de Lisboa (CAML), Faculdade de Medicina da Universidade de Lisboa, Av. Prof. Egas Moniz MB, 1649-028 Lisbon, Portugal; 2 Serviço de Cardiologia, Centro Hospitalar Barreiro Montijo (CHBM), EPE, Barreiro, Portugal; 3 Área Disciplinar Autónoma de Bioestatística (Laboratório de Biomatemática), Instituto Medicina Preventiva E Saúde Publica, Faculdade de Medicina da Universidade de Lisboa, Av. Prof. Egas Moniz MB, Lisbon, 1649-028 Portugal; 4 Departamento Do Coração E Vasos, Centro Hospitalar Universitário Lisboa Norte (CHULN), EPE, Lisbon, Portugal

**Keywords:** Infective endocarditis, Incidence, Surgery, Microbiology, Mortality, Temporal trends

## Abstract

**Background:**

Nationwide hospital admissions data series have contributed to a reliable assessment of the changing epidemiology of infective endocarditis, even though conclusions are not uniform. We sought to use a recent populational series to describe the temporal trends on the incidence of infective endocarditis, its clinical characteristics and outcome results, in Portugal.

**Methods:**

A nationwide retrospective temporal trend study on the incidence and clinical characterization of patients hospitalized with infective endocarditis, between 2010 and 2018.

**Results:**

7574 patients were hospitalized with infective endocarditis from 2010 to 2018 in Portuguese public hospitals. The average length of hospitalization was 29.3 ± 28.7 days, predominantly men (56.9%), and 47.1% had between 60 and 79 years old. The most frequent infectious agents involved were *Staphylococcus* (16.4%) and *Streptococcus* (13.6%). During hospitalization, 12.4% of patients underwent heart valve surgery and 20% of the total cohort died. After a 1-year post-discharge follow-up, 13.2% of the total initial cohort had had heart valve surgery and 21.2% in total died. The annual incidence of infective endocarditis was 8.31 per 100,000 habitants, being higher in men (9.96 per 100,000 in males versus 6.82 in females, *p* < 0.001) and increased with age, peaking at patients 80 years old or older (40.62 per 100,000). In-hospital mortality rate significantly increased during the analyzed period, the strongest independent predictors being ischemic or hemorrhagic stroke, sepsis, and acute renal failure. Younger age and cardiac surgery had a protective effect towards a fatal outcome.

**Conclusions:**

In Portugal, between 2010 and 2018, the incidence of infective endocarditis presented a general growth trend with a deceleration in the most recent years. Also, a significant rate of in-hospital complications, a mildly lower than expected stable surgical rate and a still high and growing mortality rate were noted.

**Supplementary Information:**

The online version contains supplementary material available at 10.1186/s12872-021-01937-3.

## Introduction

Infective endocarditis (IE) is an old and rare disease, with an annual incidence in populational studies of 4 to 10 cases per 100,000 per habitants [1]. Indeed, all IE cases culminate in hospitalization at some point. And despite decades of improvement in the medical and surgical field, a persistent substantial rate of complications and mortality is shown in IE. In fact, worldwide, the intrahospital mortality rate varies among different countries, varying between 8 and 40% [2]. Several factors may contribute to this variation such as the country surveyed, the duration of the research, the analyzed time, variations in the population studied, considered IE case definition and organization of the health systems.

A shift in the epidemiology of endocarditis has been noted in the most recent literature [3–5], with older and more complex patients, *Staphylococcus aureus* as an important and emerging agent, a growing susceptible adult population of patients with congenital heart disease or intracardiac prosthesis or devices and chronic renal failure on dialysis. The diagnostic criteria changed in 2000 [6] with the evolution of transesophageal echocardiography and microbiology and in 2015 [7] with the inclusion of new imaging diagnostic techniques such as 18F-FDG PET/CT. Timing of surgery and antibiotic prophylaxis continue to be challenging issues in daily practice.

Little information is known about Portugal’s temporal trends regarding IE incidence and clinical results. The main source of evidence regarding the IE situation in Portugal arises from single-center hospital surveys [8–12] and conclude that it predominantly occurs in older male patients, with a predominance of *Staphylococcus* and *Streptococcus* species and an apparently high rate of intrahospital complications. Nevertheless, several questions remain unanswered regarding epidemiological and outcome results in patients hospitalized with IE in Portugal.

Using populational-based data we aimed to understand the incidence of IE in Portugal, describe its clinical, microbiological, complications and outcome results, and analyze its evolution between 2010 and 2018.

## Methods

### Study design and data source

A nationwide retrospective temporal trend study on the incidence of IE was performed.

Data were obtained from the Central Administration of the Health System of the Portuguese Ministry of Health (Administração Central Sistemas Saúde—ACSS-). This system contains administrative and clinical data of all admissions and discharges to National Health System hospitals, which covers almost the whole population of Portugal. Until 2014, it only included Portugal Mainland public hospitals. From 2015 onwards, it included the Madeira and Azores public hospital data. Hospitalizations in private hospital centers are not included.

The hospital discharge report contains clinical information (sex, age, geographical region, hospital institution, date of admission and discharge, length of hospitalization, disposition—home, unknown, another acute hospital center, palliative care institution, outpatient clinics, discharge against medical advice, deceased), a clinical diagnosis list (a primary diagnosis and up to nineteen secondary diagnoses) and procedures (up to twenty). It is completed by the medical team that was responsible for the patient during hospitalization. Hospital discharge report coding, using the International Classification of Diseases (ICD) is then performed by specially trained medical staff, based on the medical report and other clinical documents available on the patient’s file.

Until 2016, the 9th Revision Clinical Modification (ICD-9-CM) was used to code for diagnosis and procedures. From 2016 to the present day, the 10th Revision Clinical Modification (ICD-10-CM) has been used.

### Study population and patients

The study population covered patients from all age groups, from Mainland Portugal until 2014 and from Portugal (mainland and islands Azores and Madeira) from 2015 to 2018.

In this study, we retrospectively identified all cases of incident IE episodes analyzing all hospital episodes of patients with a discharge diagnosis of infective endocarditis (ICD-9-CM codes 421.0, 421.1, 421.9 and 424.9; ICD-10-CM I33.0, I33.9, I38 and I39) between 1st January 2010 and 31st December 2018. Patients admitted in 2009 with a diagnosis of IE were removed (prevalent cases). Day case episodes were also excluded.

IE cases were recognized through the identification of all episodes of hospitalization that contained an IE compatible code as the primary or secondary diagnosis. The use of modified Duke criteria [13] has been the mainstay for the diagnosis of IE in Portugal, with a gradual integration of the newer imaging modalities since 2015 as per European scientific guidelines indication [7].To avoid overcounting, we identified the first episode of each patient with an IE compatible code for the analyzed period. From this group of patients, we identified those that were transferred to another acute care hospital (a 7.9% of the total cohort, with zero- or one-day difference between the discharge date and the subsequent admission for IE), guaranteeing combination between subsequent episode to the previous one so that all patients were considered to have a single episode/hospitalization for IE.

All subsequent hospital episodes following one year after the first hospitalization for IE were analyzed to identify hospital readmissions and the performance of cardiac surgery.

### Variables

For incident IE episodes, clinical information (sex, age, year of discharge length of hospitalization), previous heart history and comorbidities (diabetes mellitus, non-rheumatic valve disease, rheumatic valve disease, arterial hypertension, chronic renal disease, chronic coronary artery disease, cancer, HIV, cardiac devices and heart valve prosthesis, atrial fibrillation, chronic liver disease, chronic obstructive lung disease, use of opioid drugs, congenital heart disease), microorganisms (*Staphylococcus, Streptococcus, Enterococcus*, Gram-negative, anaerobes, fungus, Brucella), IE compatible complications (heart failure, embolic stroke, ischemic stroke, transient ischemic accident, septic shock, splenic abscess, acute renal failure, acute coronary syndrome, central nervous system abscess or meningitis), cardiac surgery, and intrahospital death were collected using the ICD-9 and ICD-10 codes—see Additional file 1: Table S1.

### Outcomes

For each year, the incidence of IE in Portugal was estimated as the number of new cases per year and was expressed as 100,000 inhabitants/year and was calculated as a nine-year average. Annual mainland Portuguese population until 2014 and total Portuguese population after 2014 (denominator) were obtained from census population (2011) or estimates for intercensal years, at Statistics Portugal [the Portuguese National Instituto of Statistics (INE)] database.

The annual incidence was also assessed for the age group (< 18; 18–39; 40–49; 50–59; 60–79 and ≥ 80 years old) and sex-specific groups.

The size of the population (total, sex and group age-specific) of Portugal was obtained from the information line of the National Institute of Statistics (Instituto Nacional Estatística—INE—www.ine.pt) and corresponds to the estimates of the resident population at the end of the year, for each year of the study. The Portuguese population was 10,562,178 in 2011 (National Census performed in 2011) decreasing to 10,276,617 in 2018 [14].

Among patients hospitalized with incident IE, the annual rate of in-hospital mortality was found.

From the analysis of subsequent hospital readmissions for up to one year, the rates of total cardiac surgery performed on the cohort, 30 day and 1-year hospital readmission rates and hospital death within one year of initial admission were also obtained.

### Statistics

Continuous variables were presented as mean ± standard deviation and categorical variables expressed as frequencies and percentages. The comparison between continuous variables was done through t Student’s test or Mann Whitney test, and the comparison between categorical variables was calculated with the chi-square test or Fisher exact test. Linear by Linear association was performed to test for a linear trend during the analyzed period.

To assess the factors associated with in-hospital surgical intervention and in-hospital mortality, the inferential analysis was performed using multiple logistic regression (a generalized linear model using Poisson distribution and the logit link function). The Stepwise (Forward) method based on the Akaike information criterion minimization was used for the selection of variables included in the model. The adjusted odds ratio, as well as the 95% Confidence interval (CI 95%), were estimated for each variable included in the regression model.

The temporal trend analysis of IE incidence (total, by age-group, and by gender) was performed using Linear Correlation Coefficient, linear and quadratic regression, using the year variable and its square.

All tests were 2-tailed. The level of significance was set to α = 0.05.

The data were analyzed using IBM SPSS Statistics for Windows version 24 software (IBM Corp., Armonk, NY, USA).

## Results

### Study population demographic, medical background and in-hospital outcomes

7574 patients were admitted with incident IE from 2010 to 2018 in Portugal, with 8172 episodes of hospitalization—see Table 1. The average length of hospitalization was 29.3 ± 28.7 days (minimum 1 and maximum 439 days, median 21 days). The majority were male (56.9%), and nearly half of the cohort had between 60 and 79 years old. A relevant prevalence of diabetes (26.6%), arterial hypertension (37.3%), atrial fibrillation (24.8%) and heart valve disease (30.4%) were noted.

**Table 1:** Demographic, medical background and infectious agents involved in IE hospitalizations

	2010–2018
Number of patients, n	7574
Male, n (%)	4308 (56.9)
Age, years (SD)	68.3 ± 17.3
Age groups, n (%)	
< 18	91 (1.2)
18–39	503 (6.6)
40–59	1253 (16.5)
60–79	3565 (47.1)
≥ 80	2162 (28.5)
Length of hospital stay, days (SD)	29.3 ± 28.7 Median 21
Medical background, n (%)	
Diabetes mellitus	2016 (26.6)
Arterial hypertension	2828 (37.3)
Atrial fibrillation	1876 (24.8)
HIV	133 (1.8)
CRF	887 (11.7)
CRF on Hemodialysis	324 (4.3)
Non-rheumatic cardiac valve disease	1590 (21.0)
Rheumatic valve disease	709 (9.4)
Cardiac valve prosthesis	914 (12.1)
Cardiac implantable electronic devices	649 (8.6)
Coronary artery disease	970 (12.8)
Previous PCI	108 (1.4)
Previous CABG	183 (2.4)
Congenital heart disease	42 (1.0)
Cancer	1018 (13.4)
COPD	702 (9.3)
Opioid consumption	103 (1.4)
Chronic hepatic disease	366 (4.8)
Infectious agents, n (%)	
*Staphylococcus*	1242 (16.4)
*Streptococcus*	1030 (13.6)
*Enterococcus*	535 (7.1)
Gram-negative	898 (11.9)
Anaerobes	20 (0.3)
Fungi	10 (0.1)
*Brucella*	9 (0.1)

*CABG* coronary artery bypass graft, *COPD* Chronic Obstructive Pulmonary Disease, *CRF* chronic renal failure, *HIV* human immunodeficiency virus, *PCI* percutaneous coronary intervention

A microorganism was coded in 49.5% of the incident episodes of IE. The most frequent infectious agents involved were *Staphylococcus* (16.4%) and *Streptococcus* (13.6%), followed by gram-negative bacteria (11.9%).

During the first hospitalization for IE, 3995 patients (52.7%) had an in-hospital complication. The most frequent IE related complication were heart failure (29.5%), followed by sepsis (12.4%), neurologic events (ischemic stroke affected nearly 10% of the total cohort) and acute renal failure (10.8%)—see Table 2.

**Table 2:** IE related complications, cardiac surgery and mortality during incident hospitalization for IE

	2010–2018
In-hospital complications/outcomes, n (%)	
Heart failure	2232 (29.5)
Sepsis	937 (12.4)
Ischemic CNS event	743 (9.8)
Hemorrhagic CNS event	204 (2.7)
Non-neurological systemic embolism	97 (1.3)
Splenic abscess	73 (1.0)
CNS abscess/meningitis	91 (1.2)
Acute renal failure	819 (10.8)
Acute myocardial infarct	220 (2.9)
Cardiac surgery, incident episode, n (%)	937 (12.4)
In-hospital death, incident episode, n (%)	1513 (20.0)
One-year follow-up, n (%)	6061
30-day re-hospitalization	294 (4.8)
1-year re-hospitalization	599 (9.8)
Total cardiac surgery—including 1-year follow-up, n (%)	998 (13.2)
Death—including 1-year follow-up, n (%)	1611 (21.2)

*CNS* central nervous system

During the incident hospitalization for IE, 937 (12.4%) patients received heart valve surgery.

Of the patients with incident IE, 5058 (66.8%) were discharged home, 64 (1%) were transferred to a rehabilitation or palliative care institution and in 939 patients (12.4%) the destination post discharge is unidentified (unknown or discharge against medical advice). One-fifth of the total cohort (1513 patients) died.

**Fig. 1:**
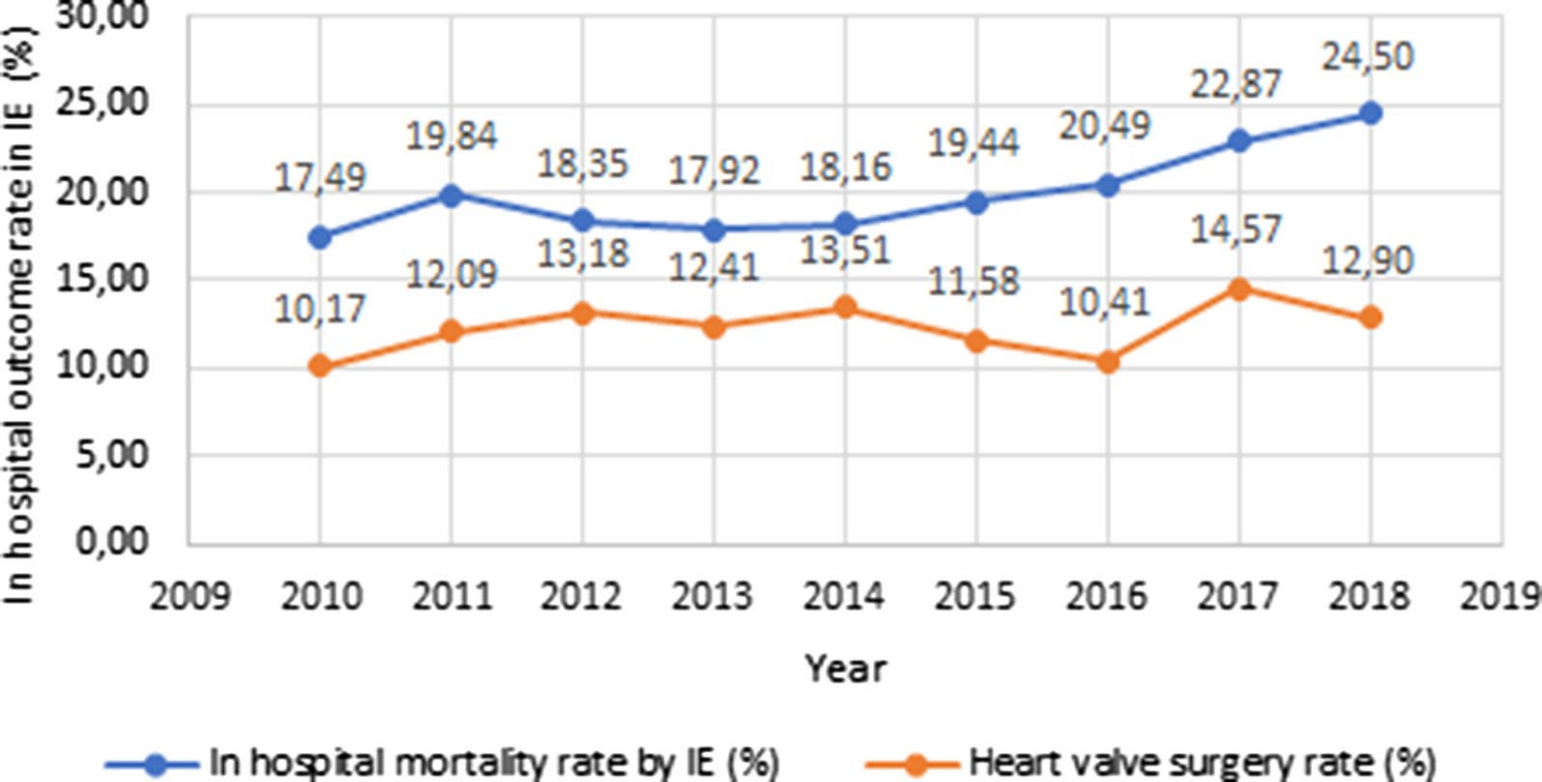
Evolution of annual in-hospital surgical rate and mortality rate (%) among patients hospitalized with IE, in Portugal, 2010–2018

During the studied period, a reduction was noted in the rate of younger patients admitted with IE (particularly between 18 and 39 years old); as the opposite, an increase was noted in the older patients (octogenarians). Also, a trend towards an increase in comorbidities such as diabetes mellitus, arterial hypertension, atrial fibrillation, chronic renal failure and cancer was noted—see Table 3. Likewise, the proportion of IE related to cardiac valve prosthesis and intracardiac devices rose. IE caused by *Staphylococcus* spp. steadily increased overtime whereas *Streptococcus* spp. presented a mild overall decline. Regarding IE-related complications, there was overall increment overtime, mainly noted with systemic embolism and acute renal failure rates. The in-hospital surgical rate remained stable during the observed period, oscillating between 10.2 and 14.6%—see Fig. 1.

**Table 3:** Progression of IE patients by clinical, microbiological and outcomes

	2010	2011	2012	2013	2014	2015	2016	2017	2018	p
Total cohort	n = 629	n = 761	n = 812	n = 854	n = 903	n = 967	n = 903	n = 892	n = 853	
Male	367	450	452	480	520	539	496	527	477	0.44
	58.3%	59.1%	55.7%	56.2%	57.6%	55.7%	54.9%	59.1%	55.9%	
< 18 years	9	11	8	7	10	9	10	12	15	< 0.001
	1.4%	1.4%	1.0%	0.8%	1.1%	0.9%	1.1%	1.3%	1.8%	
18–39 years	63	76	69	57	56	56	51	36	39	
	10.0%	10.0%	8.5%	6.7%	6.2%	5.8%	5.6%	4.0%	4.6%	
40–59 years	105	164	147	143	144	159	126	146	119	
	16.7%	21.6%	18.1%	16.7%	15.9%	16.4%	14.0%	16.4%	14.0%	
60–79 years	316	353	383	394	433	429	433	434	390	
	50.2%	46.4%	47.2%	46.1%	48.0%	44.4%	48.0%	48.7%	45.7%	
≥ 80 years	136	157	205	253	260	314	283	264	290	
	21.6%	20.6%	25.2%	29.6%	28.8%	32.5%	31.3%	29.6%	34.0%	
Diabetes mellitus	129	182	204	225	241	283	243	262	247	*0.001*
	20.5%	23.9%	25.1%	26.3%	26.7%	29.3%	26.9%	29.4%	29.0%	
Cardiac valve disease	120	159	163	174	189	212	179	224	170	0.13
	19.1%	20.9%	20.1%	20.4%	20.9%	21.9%	19.8%	25.1%	19.9%	
Rheumatic valve disease	53	74	81	79	109	94	86	74	59	0.12
	8.4%	9.7%	10.0%	9.3%	12.1%	9.7%	9.5%	8. .3%	6.9%	
Atrial fibrillation	139	178	221	230	267	301	268	138	134	< *0.001*
	22.1%	23.4%	27.2%	26.9%	29.6%	31.1%	29.7%	15.5%	15.7%	
Arterial hypertension	185	222	284	295	332	368	350	390	402	< *0.001*
	29.4%	29.2%	35.0%	34.5%	36.8%	38.1%	38.8%	43.7%	47.1%	
Chronic renal failure	49	52	43	82	88	98	114	189	172	*< 0.001*
	7.8%	6.8%	5.3%	9.6%	9.7%	10.1%	12.6%	21.2%	20.2%	
HIV	19	16	15	11	17	16	15	10	14	*0.030*
	3.0%	2.1%	1.8%	1.3%	1.9%	1.7%	1.7%	1.1%	1.6%	
Cancer	86	97	120	121	150	147	122	96	79	*0.004*
	13.7%	12.7%	14.8%	14.2%	16.6%	15.2%	13.5%	10.8%	9.3%	
COPD	62	75	74	87	109	120	85	43	47	< *0.001*
	9.9%	9.9%	9.1%	10.2%	12.1%	12.4%	9.4%	4.8%	5.5%	
Cardiac valve prosthesis	70	90	98	98	86	116	109	132	115	*0.030*
	11.1%	11.8%	12.1%	11.5%	9.5%	12.0%	12.1%	14.8%	13.5%	
Cardiac device	39	61	63	58	61	79	88	87	113	*< 0.001*
	6.2%	8.0%	7.8%	6.8%	6.8%	8.2%	9.7%	9.8%	13.2%	
CAD	60	81	107	97	130	130	122	114	129	*0.001*
	9.5%	10.6%	13.2%	11.4%	14.4%	13.4%	13.5%	12.8%	15.1%	
Chronic hepatic disease	30	41	39	45	62	47	42	28	32	*0.03*
	4.8%	5.4%	4.8%	5.3%	6.9%	4.9%	4.7%	3.1%	3.8%	
*Staphylococcus*	95	114	115	130	145	158	145	170	170	< *0.001*
	15.1%	15.0%	14.2%	15.2%	16.1%	16.3%	16.1%	19.1%	19.9%	
*Streptococcus*	83	124	114	120	149	124	125	104	87	*0.002*
	13.2%	16.3%	14.0%	14.1%	16.5%	12.8%	13.8%	11.7%	10.2%	
*Enterococcus*	40	53	57	54	74	63	75	61	58	0.60
	6.4%	7.0%	7.0%	6.3%	8.2%	6.5%	8.3%	6.8%	6.8%	
Gram negatives	57	87	98	104	132	140	123	65	92	0.69
	9.1%	11.4%	12.1%	12.2%	14.6%	14.5%	13.6%	7.3%	10.8%	
Acute MI	12	30	27	20	21	30	31	28	21	0.96
	1.9%	3.9%	3.3%	2.3%	2.3%	3.1%	3.4%	3.1%	2.5%	
HF	179	193	238	256	264	316	303	253	230	0.28
	28.5%	25.4%	29.3%	30.0%	29.2%	32.7%	33.6%	28.4%	27.0%	
Ischemic stroke	50	64	75	71	100	91	95	92	68	0.28
	7.9%	8.4%	9.2%	8.3%	11.1%	9.4%	10.5%	10.3%	8.0%	
Hemorrhagic stroke	11	18	24	20	31	22	25	34	19	0.24
	1.7%	2.4%	3.0%	23%	3.4%	2.3%	2.8%	3.8%	2.2%	
Systemic embolism	2	1	4	8	2	8	15	31	26	< *0.001*
	0.3%	0.1%	0.5%	0.9%	0.2%	0.8%	1.7%	3.5%	3.0%	
Splenic abscess	4	11	10	10	16	11	9	1	1	*0.006*
	0.6%	1.4%	1.2%	1.2%	1.8%	1.1%	1.0%	0.1%	0.1%	
Acute renal failure	68	88	63	78	80	70	81	141	150	< *0.001*
	10.8%	11.6%	7.8%	9.1%	8.9%	7.2%	9.0%	15.8%	17.6%	
Sepsis	64	102	113	101	128	142	102	108	108	0.88
	10.2%	13.4%	13.9%	11.8%	14.2%	14.7%	11.3%	12.1%	12.7%	
In hospital IE related complications	305	381	428	435	479	540	504	478	445	*0.01*
	48.5%	50.1%	52.7%	50.9%	53.0%	55.8%	55.8%	53.6%	52.2%	
Cardiac valve surgery	64	92	107	106	122	112	94	130	110	0.25
	10.2%	12.1%	13.2%	12.4%	13.5%	11.6%	10.4%	14.6%	12.9%	
Death	110	151	149	153	164	188	185	204	209	< *0.001*
	17.5%	19.8%	18.3%	17.9%	18.2%	19.4%	20.5%	22.9%	24.5%	

*CAD* Coronary Artery Disease, *COPD* chronic obstructive pulmonary disease, *HF* heart failure, *HIV* human immunodeficiency virus, *MI* myocardial infarction

Values in Italic highlight *p* values < 0.05

599 patients were readmitted during the first-year post-discharge after an incident episode of IE (9.8% of the total cohort that survived the first hospitalization)—see Table 2. More than half of these patients were readmitted during the first 30 days post-discharge. During the first year follow up 61 more patients had heart valve surgery totalizing 13.2% of the total cohort and 98 patients died (totalizing 21.2% of the total initial cohort).

### Incidence rate and temporal trend of IE in Portugal

In Portugal, from 2010 to 2018, the incidence of IE varied between 6.25 cases and 9.35 per 100,000—see Table 4. The average annual incidence was 8.31 per 100,000 inhabitants (95% CI 7.59–9.03).

**Table 4:** Incidence evolution of IE, by total cohort, by age, and by sex

	Total	Age group (years)					Sex	
		< 18	18–39	40–59	60–79	≥ 80	Male	Female
2010	6.25	0.38	2.16	3.73	15.78	27.10	7.64	4.99
2011	7.59	0.47	2.66	5.80	17.45	29.97	9.40	5.93
2012	8.14	0.35	2.48	5.18	18.76	37.85	9.51	6.89
2013	8.61	0.31	2.11	5.02	19.16	45.19	10.18	7.19
2014	9.15	0.45	2.13	5.04	20.86	45.04	11.11	7.38
2015	9.35	0.38	2.06	5.26	19.69	51.10	11.00	7.87
2016	8.76	0.43	1.92	4.14	19.71	44.71	10.16	7.50
2017	8.67	0.52	1.38	4.78	19.50	40.81	10.83	6.73
2018	8.30	0.66	1.51	3.89	17.32	43.84	9.83	6.93

Incidences are reported per 100,000 inhabitants

**Fig. 2:**
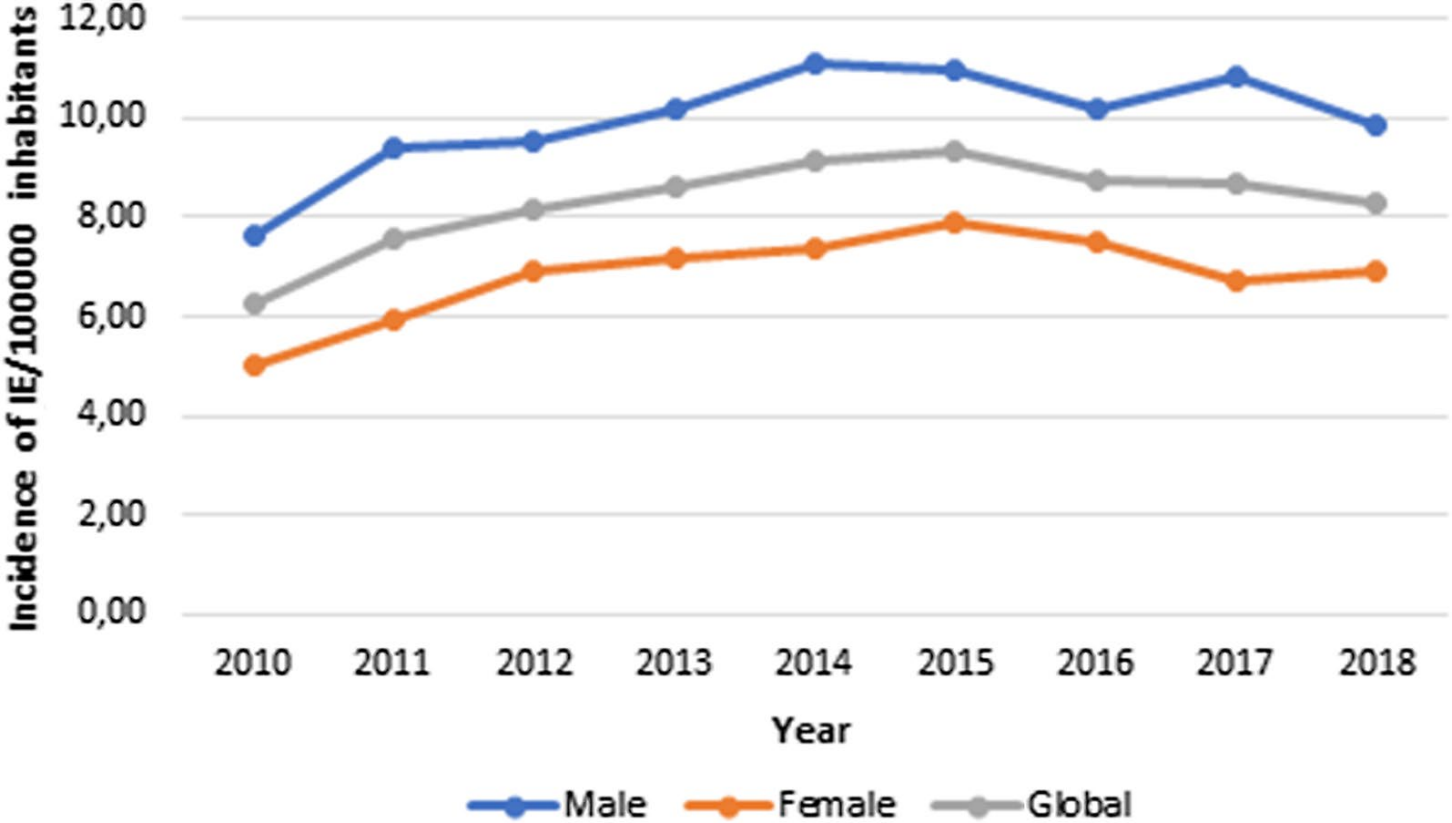
Global and sex-specific annual incidence of IE evolution, in Portugal, from 2010 to 2018

IE was more frequent in males [9.96 per 100,000 (95% CI 9.14–10.78) versus 6.82 (95% 6.15–7.51) in females, p < 0.001], with an annual ratio male/female varying between 1.3 and 1.6—see Table 4 and Fig. 2.

**Fig. 3:**
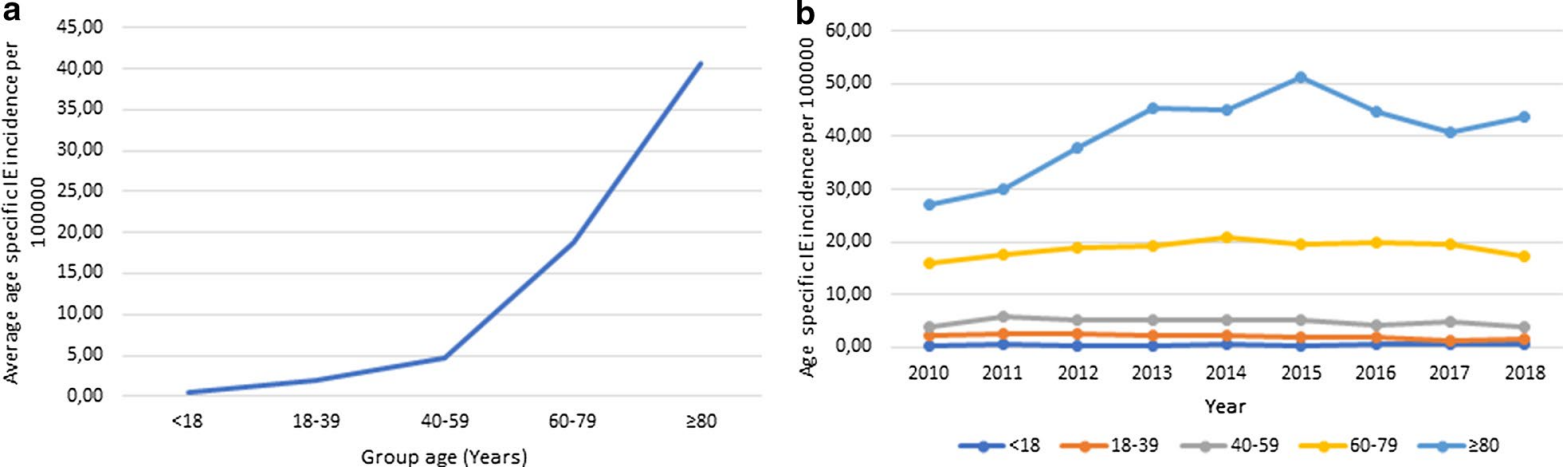
Nine-year period average age-specific incidence of IE (**a**) and group age-specific annual incidence of IE (**b**) per 100,000 habitants, in Portugal, from 2010 to 2018

This incidence also was higher with age, peaking at patients in the oldest age group (with 80 years old or older) [40.62 per 100,000 (95% CI 34.66–46.59)]—see Table 4 and Fig. 3a.

Using Pearson’s linear correlation coefficient and linear and quadratic regression analysis on the global incidence of IE in Portugal during the nine years, a globally upper trend was noted with a maximum value occurring in 2015 (9.35 per 100,000 inhabitants)—a deceleration was noted afterward.

The same trend was observed regarding sex-specific incidence in males and females and older patients (≥ 40 years old). Regarding younger patients, a declining tendency is noted quite early (from 2011 onwards)—see Fig. 3b.

### Prediction factors related to hospital mortality in IE

IE-specific in-hospital mortality rate significantly increased from 2010 (17.5%) to 2018 (24.5%)—see Fig. 1 and Table 3.

Logistic regression analysis for in-hospital mortality is summarized in Table 5. All 27 variables stated in the first column were available for analysis for all individuals. Independent prognosticators of in-hospital mortality were the presence of previous cardiac valve disease, cancer, chronic renal or hepatic disease, HIV, *Staphylococcus* or Gram-negative infection, acute myocardial infarction, heart failure, older age (specifically after 80 years old), ischemic or hemorrhagic stroke, sepsis and acute renal failure, the last were the strongest predictors. Younger age (particularly less than 40 years old) was a protective factor regarding fatal outcome. Cardiac surgery also had a protective effect on in-hospital mortality—see Table 5—odds ratio = 0.69 (95% CI 0.55–0.86, *p* = 0.001).

**Table 5:** Logistic regression analysis of in-hospital mortality

	Bivariate analysis				Full model				Stepwise model			
	OR	95% CI		*p*	Adjusted OR	95% CI		*p*	Adjusted OR	95% CI		*p*
Age group, years old												
70–79	0.906	0.797	1.030	0.131	0.793	0.685	0.918	*0.002*	0.806	0.697	0.931	*0.003*
60–69	0.683	0.570	0.815	< *0.001*	0.519	0.418	0.644	< *0.001*	0.528	0.427	0.652	< *0.001*
40–59	0.257	0.178	0.361	< *0.001*	0.182	0.119	0.272	< *0.001*	0.186	0.122	0.276	< *0.001*
18–39	0.114	0.028	0.304	< *0.001*	0.117	0.028	0.327	< *0.001*	0.117	0.028	0.327	< *0.001*
Length of stay (days)	0.998	0.996	1.000	*0.037*	0.989	0.986	0.992	< *0.001*	0.990	0.987	0.992	< *0.001*
Previous medical history												
Cardiac valve disease	1.192	1.041	1.363	*0.010*	1.234	1.052	1.446	*0.010*	1.234	1.051	1.445	*0.010*
Rheumatic valve disease	1.241	1.030	1.489	*0.021*	1.188	0.962	1.460	0.106	1.186	0.961	1.456	0.107
Cardiac devices	1.135	0.931	1.375	0.5	0.994	0.798	1.231	0.957				
Arterial hypertension	0.740	0.656	0.834	< *0.001*	0.703	0.612	0.807	< *0.001*	0.705	0.614	0.808	< *0.001*
Atrial fibrillation	1.200	1.056	1.362	*0.005*	0.929	0.803	1.073	0.318				
Diabetes mellitus	1.275	1.126	1.442	< *0.001*	1.056	0.916	1.215	0.453				
HIV	1.618	1.094	2.346	*0.013*	2.548	1.614	3.958	< *0.001*	2.554	1.619	3.961	< *0.001*
Chronic renal failure	1.685	1.436	1.973	< *0.001*	1.230	0.995	1.515	0.054	1.298	1.081	1.555	*0.005*
Chronic renal failure on hemodialysis	1.679	1.307	2.142	< *0.001*	1.143	0.824	1.580	0.420				
Cancer	1.215	1.035	1.422	*0.016*	1.271	1.065	1.513	*0.007*	1.277	1.070	1.520	*0.006*
Chronic hepatic disease	1.753	1.386	2.204	< *0.001*	1.623	1.239	2.112	< *0.001*	1.628	1.243	2.118	< *0.001*
Infectious agents												
*Staphylococcus*	2.314	2.021	2.647	< *0.001*	1.836	1.551	2.171	< *0.001*	1.825	1.547	2.151	< *0.001*
*Enterococcus*	1.199	0.968	1.475	0.090	1.187	0.931	1.505	0.161				
Anaerobes	2.162	0.811	5.289	*0.101*	1.748	0.591	4.766	0.289				
Gram negative	1.487	1.264	1.744	< *0.001*	1.215	1.008	1.460	*0.039*	1.220	1.012	1.465	*0.035*
In-hospital complications												
Acute myocardial infarct	2.361	1.777	3.117	< *0.001*	1.899	1.382	2.591	< *0.001*	1.908	1.388	2.604	< *0.001*
Heart failure	1.503	1.335	1.693	< *0.001*	1.445	1.260	1.657	< *0.001*	1.424	1.245	1.629	< *0.001*
Ischemic stroke	2.198	1.857	2.597	< *0.001*	2.265	1.871	2.737	< *0.001*	2.259	1.868	2.726	< *0.001*
Transient ischemic accident	0.111	0.006	0.512	*0.030*	0.118	0.007	0.562	*0.037*	0.121	0.007	0.577	*0.039*
Hemorrhagic stroke	3.299	2.483	4.371	< *0.001*	3.933	2.848	5.414	< *0.001*	3.893	2.823	5.354	< *0.001*
Acute renal failure	3.378	2.899	3.933	< *0.001*	2.651	2.221	3.162	< *0.001*	2.641	2.214	3.146	< *0.001*
Systemic embolism	1.810	1.157	2.766	*0.007*	1.014	0.590	1.703	0.960				
Central nervous system abscess/meningitis	1.442	0.884	2.273	0.127	0.982	0.556	1.687	0.948				
Sepsis	5.868	5.088	6.769	< *0.001*	4.673	3.969	5.505	< *0.001*	4.724	4.016	5.560	< *0.001*
Cardiac surgery	0.712	0.589	0.855	< *0.001*	0.689	0.547	0.864	*0.001*	0.692	0.550	0.867	*0.001*

Age group reference: 80 +; for medical history conditions, infectious agents, in-hospital complications, and cardiac surgery reference categories were: “Absence/No”

*CI* confidence interval, *HIV* human immunodeficiency virus, *OR* odds ratio

Values in Italic highlight *p* values < 0.05

## Discussion

Our study identified 7574 patients with incident IE hospitalized between 2010 and 2018, in Portugal. Four main extrapolations were obtained from this analysis. First, the annual crude populational incidence of IE in Portugal averaged 8.3 cases per 100,000 habitants (95% CI 7.59–9.03), with a general growing trend. Second, *Staphylococcus* was the most prevalent infectious agent in this study. Third, the surgical intervention rate was slightly lower than expected but its protective effect towards prognosis was demonstrated. Finally, this recent analysis confirms that despite all efforts to improve its prognosis, a still too high mortality was noted, with a rising trend.

Incidence estimation can only be performed using populational based data. With regards to IE, populational surveys are scarce and with significant variations regarding methodology and results. The reported incidence of IE among different studies is not entirely similar ranging between 3 and 15 cases per 100,000 in population-based studies [15–27], with considerable differences noted even in similar countries [28]. Still, the findings in Portugal are consistent with the already described values. Additionally, an upper trend in incident IE was noted between 2010 and 2018. The strength of this observation is probably restricted by the fact that we are analyzing a short period. Nevertheless, this has been observed in several developed countries such as Denmark, Italy, England, Spain, Germany, or the Netherlands in the past two decades. This tendency was not noted, however, in other countries such as France, Australia, Scotland or the United States of America (USA) [16, 20]. Reasons to explain this global upper trend in IE incidence in Portugal are various, although some may be speculative as this study does not allow for a cause-effect analysis. Portugal has experienced a gradual increase in life expectancy of the population [29], population aging [30] with a sustained increase in the aging index of the population [31]. This, adding to a growing proportion of patients with comorbidities associated with an increased rate of invasive medical procedures (cardiac and non-cardiac) can partially explain the observed tendency. Of course, the improvement in microbiology, imaging techniques and medical expertise (clinician’s awareness and technical specialization) that are available in the public health system to diagnose IE can also contribute to the observed trend in developed countries.

Regarding microbiology, the data retrieved from discharge notes were scarce (49.5% of the total cohort), probably a result of underreporting—this has been noted in other populational studies such as in Fedeli et al. [18] or Shah et al. [27]. Infectious agents such as *Staphylococcus* spp. and *Streptococcus* spp. were the most prevalent in this analysis, with a mild predominance of the former, which is according to other tertiary hospitals series [18, 29, 32]. In fact, in developed countries *Staphylococcus* has replaced *Streptococcus* as the most prevalent infectious agent causing IE in the last decades, mainly justified by an increase in invasive medical procedures in the general population as well as a significant population of IV drug use. Our data does not allow for analysis regarding the source of IE (community versus healthcare-associated). On the other hand, the low percentage of patients with opioid consumption or HIV in our cohort is probably related to underreporting or underdiagnosis at the time of hospitalization. Unpredictably, gram-negative bacteria were the third most frequent cause of IE in our study (11.9%), followed by *Enterococcus*.

The increasing role of cardiac surgery in IE management is well documented in all scientific guidelines [7, 33]. The surgical rate in other populational studies ranged from 15 to nearly 50%; yet, the most recent populational series that included patients hospitalized after 2010 reported heterogeneous surgical rates of 10.6–13.3% (Toyoda et al. [24], USA), 3.8–6.3% (Shah et al. [27], Scotland), 23% (Olmos et al. [26], Spain) and 46.5% (Cresti et al. [25], Italy). Our analysis presented marginally lower values of surgical rate (up to 13.2% at one-year follow-up had had valve surgery), which may be a consequence of an older cohort, with significant comorbidities and a high rate of noncardiac complications as has been described individually in Portuguese single-center surveys [8]. Also, a part of the total cohort may have had surgery after discharge from the incident hospitalization and the IE code may have been underreported in the surgical hospitalization report with loss of that data during this evaluation. An important aspect of our analysis was the demonstration of a clear clinical benefit of surgery which was also noted in the ICE registry [32], in a metanalysis by Head et al. [34] and in other tertiary referral centers surveys [10, 11, 35, 36]. Still, a general described temporal trend is on stability [18, 20, 32, 37], which is consistent with our data, despite more recent series reporting an increasing tendency in Spain [26] and the USA [38].

One-fifth of the cohort died during the index hospitalization. This is in accordance with other already mentioned populational series. Trend analysis revealed an upper tendency consistent with Italian scenario [18, 25] whereas in Denmark [15], Spain [26] and the USA [38] a decrease was registered. Half the cohort presented related complications, being heart failure the most prevalent situation. An increase in the rate of prognostic factors such as older age, comorbidities (cancer or renal chronic disease), infection by *Staphylococcus* and the presence of IE related in-hospital complications may justify a higher complexity in IE cases that could thus partially explain this higher mortality during the observed period. This once again confirms the malignant nature of this disease—in fact, the multisystemic nature of the presented complications (cardiac, infectious, neurological, renal) is an argument in favor of having a multidisciplinary team of specialists managing this disease in dedicated centers to improve its prognosis at an individual level.

Finally, nearly 10% of the surviving cohort after the first hospitalization with IE were readmitted during the first year. Few studies have analyzed this specific feature. Morita et al. [39] concluded that nearly 25% of their cohort were readmitted during the first 30 days post-discharge. The fact that most of our patients were discharged home or to rehabilitation or palliative care institutions and that the length of hospital stay was considerably higher in our study (median 21 days in our study versus 10 days at Morita et al. study) may have contributed to this disparity.

The general growing incidence of IE in a contemporary cohort in Portugal, the need for prolonged hospitalization, with a significant rate of complications and mortality highlight that IE should be a clinical and national priority.

### Limitations

Our study has several limitations.

First, our data were obtained from ICD coded information obtained from discharge reports. Regarding the medical information included in the discharge report, its accuracy could not be verified at an individual patient level and therefore incomplete or erroneous information could have been included, which could have contributed to an overestimation of the IE cases. Regarding the ICD coding that was transferred for an administrative dataset, in Portugal, this process is performed by specifically trained physicians and submitted for an external audition for quality assurance. Still, coding errors or misclassifications may have occurred.

Second, as the study was retrospective and based on administrative data, identification of specific clinical and surgical factors or confounders (origin of IE: community versus health care, frailty index, antibiotic coverage, formal clinical indication for surgery, etc.) that could have a role in explaining some of the shifts observed regarding incidence, surgical rate or in-hospital mortality was limited. Also, the underreporting of relevant concomitant diagnoses could have taken place.

Third, the population of Madeira and Azores islands was not included in this analysis between 2010 and 2014 and the information regarding private hospitals was not included in this analysis which may have underestimated the number of real IE cases.

Fourth, we assumed that the infectious agents identified through the ICD coding were the causative agents of IE but this information was not validated individually. Also, half the patients did not have information on the infectious agent either because it was a negative blood culture IE or because it was underreported, and this was unfeasible to ascertain.

Finally, a selection bias for surgical intervention could have been present as high-risk patients with an indication for surgery but refused or that died before surgery were not identified.

## Conclusions

Between 2010 and 2018 in Portugal, the incidence of infective endocarditis demonstrated a global increase with a deceleration in more recent years, a significant rate of in-hospital complications, a mildly lower than expected stable surgical rate and a still too high and growing mortality rate.

The need to analyze and eventually improve existing advanced clinical protocols, hospital circuits for these patients, and the specialization of local multidisciplinary clinical teams is warranted. Additionally, optimization of national healthcare organization through the definition of specific national referral centers should be sought. Lastly, a better understanding of epidemiology is crucial in justifying and promoting favorable national health policies. This can be achieved through the implementation of a national registry that can prospectively describe IE´s diagnosis and management, the promotion of patients and physicians´ education/awareness programs, and the active intervention on risk factors that are amenable to be altered.

## Supplementary Information

**Additional file 1: Table S1.** ICD-9 and ICD-10 codes used to identify infective endocarditis cases and associated factors. Table that contains all the ICD-9 and ICD-10 codes used to identify the different diagnosis codes on the database.

## Data Availability

The data that support the findings of this study are available from *ACSS* but restrictions apply to the availability of these data, which were used under license for the current study, and so are not publicly available. Data are however available from the authors upon reasonable request and only after ACSS’s permission.

## Abbreviations

CAD: Coronary Artery Disease
CI: Confidence interval
CNS: Central nervous system
COPD: Chronic obstructive pulmonary disease
IE: Infective endocarditis
HF: Heart failure
HIV: Human immunodeficiency virus
MI: Myocardial Infarction
OR: Odds ratio
