# Supplementary material for: Insight into the epidemiology of infective endocarditis in Portugal: a contemporary nationwide study from 2010 to 2018
Source: BMC Cardiovasc Disord. 2021 Mar 16;21:138. doi: 10.1186/s12872-021-01937-3 (PMC7962378; doi:10.1186/s12872-021-01937-3)
Supplement: Supplementary file 1 — Additional file 1: Table S1. ICD-9 and ICD-10 codes used to identify infective endocarditis cases and associated factors. Table that contains all the ICD-9 and ICD-10 codes used to identify the different diagnosis codes on the database. [file 12872_2021_1937_MOESM1_ESM.pdf]

Appendix

Table S1. ICD-9 and ICD-10 codes used to identify infective endocarditis cases and associated factors.

	ICD9	ICD10
**Infective endocarditis**	421.0<br>421.1<br>421.9<br>424.9	I33.0<br>I33.9<br>I38<br>I39
**Previous medical history**		
**Diabetes mellitus**	250	E10<br>E11<br>E12<br>E13<br>E14
**Cardiac valve disease**	424.0 (Mitral V)<br>424.1 (Aortic V)<br>424.2 (Tricuspid V)<br>424.3 (Pulmonary V)	I34 (Mitral V)<br>I35 (Aortic V)<br>I36 (Tricuspid V)<br>I37 (Pulmonary V)
**Rheumatic disease**	394 (Mitral V)<br>395 (Aortic V)<br>396 (Mitral and aortic V)<br>397 (Tricuspid and Pulmonary valves)<br>398 (Other rhematic heart disease)	I05 (Mitral V)<br>I06 (Aortic V)<br>I08 (Mitral and aortic V)<br>I07 (Tricuspid and Pulmonary valves)
**Atrial fibrillation**	427.31	I48.91
**Arterial hypertension**	401	I10<br>I11
**HIV**	042<br>V08<br>07953	E93727<br>D1286<br>C836<br>B20-B24
**Chronic renal failure**	585 (5850 – 5856)<br>586	N18 (N181-N189)
**Chronic renal failure on hemodialysis**	5856<br>V451<br>V4511	N185
**Cancer**	140-239	C00-C97
**COPD**	490-496	J44.9
**Congenital heart disease**	7460-7469	Q20.0-Q20.4, Q21.2-Q21.8, Q26.2
**Use of opioid**	30400-30403 (opioid use)	F11.1-F11.2 (opioid use)
**Cardiac valve prosthesis**	V433<br>99602<br>99671	Z952<br>Z953<br>Z954<br>Z959
**Cardiac devices**	99601<br>99604<br>99672<br>V5331<br>V4501	Z95.0<br>Z95810<br>T827

**Chronic coronary artery disease**	414	I25
**S/P PCI**	V45.82	Z95.5
**S/P CABG**	V45.81	Z95.1
**Chronic hepatic disease**	571	K70<br>K74
**Microorganisms**		
**Staphylococcus**	0411<br>04111<br>04112<br>04119<br>0381<br>03810<br>03811<br>03812<br>03819	A41.0<br>A41.1<br>A41.2<br>A49.0<br>A4101<br>A4102<br>Z22321<br>Z22322<br>B95.6<br>B95.7<br>B95.8
**Streptococcus**	041.0<br>0380<br>0382	A40.0, A40.1, A 40.2, A40.3, A40.8, A40.9<br>A49.1<br>B95.0, B951, B953, B954, B955<br>Z2233
**Enterococcus**	041.04	A41.81<br>B952
**Gram negative**	03840<br>04180<br>04185<br>03841<br>03842<br>03843<br>03844<br>03849<br>0413<br>0414<br>0415<br>0416<br>0417	A41.5<br>A4150<br>A4151<br>A4152<br>A4153<br>A4159<br>B962<br>B963<br>B964<br>B965
**Anaerobes**	48281<br>04184<br>0383<br>04182<br>04183	A41.4
**Fungus**	11281<br>1160	B37.6<br>B40
**Brucella**	023	A23
**Complications**		
**Acute Myocardial Infarct**	410	I21
**Heart failure**	4280	I50.0

	4281<br>4289	I50.1<br>I50.9
**Ischemic stroke**	433<br>434	I63
**Transient ischemic accident**	435<br>4359	G459
**Hemorrhagic stroke**	430<br>431	I60<br>I61<br>I62
**Non specified stroke**	436<br>437	I64
**Systemic embolism**	449	I74.0-I74.9
**Splenic abscess**	28959	D73.3
**Central nervous system abscess/meningitis**	320<br>3200<br>3201<br>3203<br>32081<br>32082<br>32089<br>3209<br>11283<br>1142<br>11501<br>11511<br>3240<br>3241<br>3249	G00X<br>G01X<br>Go2X<br>G03X<br>G060
**Acute renal failure**	584 (5845-5849)	N17
**Sepsis**	99591<br>99592<br>78552	A40<br>A41.0-A41.4<br>A41.8<br>R6521
**Cardiac surgery**	350.x – Closed valvotomy<br>351.x – Repair<br>352.x - Replacement	**Replacement:**<br>02RF<br>02RG<br>02RH<br>02RJ4<br>**Repair**:<br>02QF<br>02QG<br>02QH<br>02QJ
